# Epidemic of Small-Pox in Quebec, Supposed to Depend upon the Opening of an Intramural Small-Pox Cemetery, 214 Years Old

**Published:** 1855-06

**Authors:** George D. Gibb

**Affiliations:** Member of the Canadian Institute.


### EPIDEMIC OF SMALL-POX IN QUEBEC, SUPPOSED TO DEPEND UPON THE OPENING OF AN INTRAMURAL SMALL-POX CEMETERY, 214 YEARS OLD.

By GEORGE D. GIBB, M.D., F.G.S., Member of the Canadian Institute.

THE extraordinary severity of the outbreak of cholera in London during the past year, in St. James's parish, accompanied with very great mortality, must be familiar to most minds. One of the causes to which this was attributed was the disturbance of the soil, for the purposes of sewerage, over the burial-ground which contained the bodies of those who died of the plague in 1665. Whether this actually was a cause is, for the present, a matter of doubt; as my friend, Dr. Snow, has shown in his work on cholera that the soil was disturbed some months previous to its invasion, and he believes that it had nothing whatever to do with the severity of the disease.

It is a matter of some moment to remember, however, that the derangement and disturbance of a peculiarly impregnated soil, such as this was, would, in some degree, affect the sewers in the immediate neighbourhood, and, most probably, the water of the different pumps used for the purposes of drink. Under such circumstances, the poisoned water and the noxious emanations from sewers might very reasonably be expected to favour the development and spread of such a disease as cholera.

There is a point of great practical importance—more important, indeed, than many persons might feel disposed to consider it—which appears to have been almost entirely overlooked in the consideration of questions relating to disturbed soils, but which must influence, to some extent, both the origin and spread of infectious diseases (at least in such peculiar instances as the present), and which hence deserves much more attention than has hitherto been bestowed upon it,—I allude to the *nature of the soil disturbed.*

We may most reasonably assume that, among the great variety of tertiary deposits, known familiarly under the name of soils, according as they approach the surface, some, such as most of the clays and some of the sands, possess the power of retaining animal matter in a state of decomposition for any length of time; and that a century or two cannot be fairly considered as remote, when, *cæteris paribus,* we look back upon the time that has elapsed, since many of the organic remains which are found at the present day were imbedded in many of these different superficial deposits. To take one or two familiar examples—I have found bivalve shells of the *Mytilus edulis,* or common muscle, in the marine tertiary clays and sands of the island of Montreal; in the latter the colour was preserved almost as perfectly as when originally deposited from the ancient sea; in the former the colour was to some extent retained, but the shells were exceedingly friable and soft, so soft, indeed, as to be preserved and procured with difficulty. Occasionally, also, shells found in the London clay possess some traces of their original colouring. These are examples of the power possessed by some soils of retaining organic colouring matter for an indefinite lapse of time; and why not, under certain conditions, animal matter?

On the other hand, gravel and analogous soils, such as a mixture of sand and gravel, or those, again, known as arenaceous, do not possess the conservative properties of those just mentioned; but owing to their filtering and drying nature they are better fitted in a sanitary point of view, to contain soft organic matter impregnated with aqueous solutions of any kind. The following quotation is taken from Dr. Snow's work, as aptly illustrating the truth of this observation. "I have been informed by an eminent engineer, that whilst a cesspool in a clay soil requires to be emptied every six or eight months, one sunk in the gravel will often go for twenty years without being emptied, owing to the soluble matters passing away into the land-springs by percolation." (p. 53.) Dr. Snow, previously to this, mentions his inability to explain whether the impurities of the pump water in St. James's parish arose from the sewers, drains, or cesspools. My own impression is, they arose from the disturbed burial-ground, which, on inquiry, I have ascertained to consist of a superficial stratum of sand and gravel over a deeper one of clay, the up-turning and mixing of which would permit of a certain amount of percolation and subsequent contamination of the water.

If the fact can be established, that some soils—either clay or sand, or a mixture of the ordinary drift with clay—do retain organic matter, and that this matter should be presented to these soils in such quantity as to offer a chance of retention for a period of time not too remote, say even three or four centuries, then are we justified in assuming that interments of persons in aggregated numbers, dying from peculiarly noxious and infectious diseases, are prone to generate either the same diseases or to spread such other contagious diseases as may be epidemic at the time, on the thorough disturbance, for any purpose whatsoever, of the soil containing them.

With a view of furnishing additional information upon the question, whether the disturbance of *old* burial grounds is likely to prove a source of danger, in giving rise to, as well as encouraging, the spread of already existing epidemic and contagious diseases, I am induced to send a few extracts from a paper published by my friend, Dr. Marsden, of Quebec, in the January number of the *Montreal Medical Chronicle*, on small pox and vaccination, more particularly as this periodical may not be within the reach of many persons in this country.

It would appear from Dr. Marsden's paper, that small-pox, in all its forms, prevailed in the city of Quebec in the last quarter of the year 1854 to an alarming extent, "so much so, indeed, as almost to give it an epidemic character". Of the truth of its being epidemic, I have no doubt whatever on carefully reading his paper, as he states particularly that he had not, in the course of the previous quarter of a century, seen so much small pox as during the past four months.

He says: "All ages and all classes have suffered, from the soldier in the garrison to the senator at his parliamentary post, and the humble citizen at his fireside; but the young of all classes have suffered most severely."

What now follows is of some importance, as furnishing evidence of the epidemic commencing in the immediate vicinity of the cemetery, which must have been very much disturbed indeed, as it was for the double purpose of sewerage and water-works, which, in a city so peculiarly built as Quebec is, must have been a matter of no ordinary difficulty.

"Among the causes assigned for this invasion of sickness, whether founded or not (and this is a point for the serious consideration of the pathological inquirer), is the opening up of the old intramural cemetery, known as the *Cimetière des Picotées*, for the desirable purposes of sewerage and water-works. How far this circumstance may have originated or contributed to the extension of the disease, I do not pretend to say; or whether the workmen employed may have carried it home to their families in different parts of the city and suburbs, but will merely state the fact that in the immediate vicinity of this burial ground, after it was broken up for the purposes beforenamed, to a depth considerably below the level employed for interments, small-pox broke out *in its vicinity*, and was more severe and fatal there than in other parts of the city and suburbs—equally among the rich and the poor; and that among the military, the first cases that occurred were from Hope Gate guardhouse, which is also in the vicinity."

That this cemetery was one used exclusively for fatal cases of small-pox, during two epidemics, is a fact well known to those versed in the records of the history of Quebec, and is one of some importance to establish in connection with an outbreak of the same disease.

"It is now 214 years since this cemetery was opened as a small-pox burial ground, during the severe and fatal visitation of the disease in 1650, where it appears, by an extract from *l'Histoire de l'Hotel-Dieu*, written by a nun, that small-pox was so prevalent, that the hospice wards could not contain all the cases, and a piece of ground adjoining it was fenced in with pickets, and bark huts erected within the enclosures, to receive the sick Indians, who were severe sufferers. Again, the same authority says, in 1708, 'this sad disease is ravaging the whole of New France. There is not a house free from it in the town. Those whose health has been preserved do not suffice to attend upon the sick. Each day the bodies of the dead are conveyed into the church in the lower town, or into the cathedral, without any ceremony, and in the evening they are buried together, sometimes as many as fifteen, sixteen, and eighteen.' And she adds, 'this has lasted many months,' &c."

So far, then, it is a well-established fact, that a cemetery was exclusively devoted for the repose of the bodies of the Indians who died of small-pox; and as far as the experience of writers on the subject goes, it appears that this disease is unusually severe and fatal among most of the aboriginal tribes throughout the world when once it has broken out; next, we find that the soil is disturbed to a depth below the level of the interments, and *is followed* by an eruption of the disease commencing *in the vicinity* of the cemetery, and thence speading, and becoming of unusual severity.

Now the question of importance to be entertained here, the answer to which will help to solve in some measure the origin of the epidemic, is, what was the nature of the soil in which this cemetery was situated? Quebec is well known to be divided into an upper and a lower town; the upper, which is strongly fortified, is built upon the limestone rocks of the lower Silurian formation, whilst the lower town covers the accumulations of clay and sand and gravel, which at one time formed the bed of the river St. Lawrence, and now overlies strata composed of the older palæozoic rocks. The gravel here is superficial, but the clay and sand beneath are intermingled, forming an argillaceous earth, with a considerable preponderance of the clay, thus constituting a soil well adapted for preserving organic animal matter and its compressed gases for an indefinite period of time, until opportunities might be afforded for their escape, by a disturbance carried below the level of a bicentenary repose. This small-pox cemetery being composed of such soils, offered a favourable medium for preserving the peculiar virus of this disease combined with animal matter; and if its contagious principles *can* remain dormant for any length of time in soils not affected by the air, nor in a percolating condition, the question, as far as the evidence goes, would appear to be answered in the affirmative, that the epidemic of small-pox broke out on the disturbance of a small-pox cemetery, where the bodies of those who died exclusively of the disease were so favourably disposed in the clayey soil as to permit of emanations and odours due to the disease itself, and hence its regeneration and propagation.

Numerous instances are recorded in Mr. Walker's work on graveyards, of the highly deleterious effects produced by escaping gases from coffins which had lain tranquil for very many years; but these cases would appear to have been the result of pent-up chemical decomposition, and not due, so far as can be known, to regenerated miasmata. Now, whether the epidemic in the present instance was the result of regeneration from variolous gaseous emanations, whether an accidental coincidence, or whether, again, due to causes remote from either of these, is a matter for study to those who have paid special attention to such matters; but I will merely observe, in conclusion, that the evidence is very strong indeed in favour of its being the consequence of the first,—variolous emanations,—an opinion which is materially strengthened by a consideration of the nature of the soil.

Without extending the limits of this paper, there arises another question out of the foregoing, which may be mentioned here without stopping to attempt a solution, although, perhaps, partially solved from what has been already said, but still worthy of future consideration; it is—whether contagious diseases really have the power of propagating themselves when opportunities are afforded for the escape of their peculiar virus, which may have lain dormant under certain circumstances for many years? This, I believe, has never before been fairly worked out, most probably from the non-occurrence of peculiar instances to draw attention to the subject. There is now ample reason to entertain it, as it arises especially in the course of the present paper.

In preparing this short communication I have felt, that any facts bearing in the slightest degree upon questions of such vital importance as have presented themselves must prove valuable, and I have, therefore, had no hesitation in bringing them before the readers of THE JOURNAL OF PUBLIC HEALTH.

59, *Guildford-street, Russell-square,*
*June* 14, 1855.

